# Novel deletion alleles of a *C. elegans* gene Y48E1C.1, named as *tm5468*, *tm5625* and *tm5626*

**DOI:** 10.17912/W2CQ14

**Published:** 2017-10-03

**Authors:** Sayaka Hori, Yuji Suehiro, Sawako Yoshina, Shohei Mitani

**Affiliations:** 1 Department of Physiology, Tokyo Women’s Medical University School of Medicine, Shinjuku-ku, Tokyo, 162-8666, Japan

**Figure 1.**
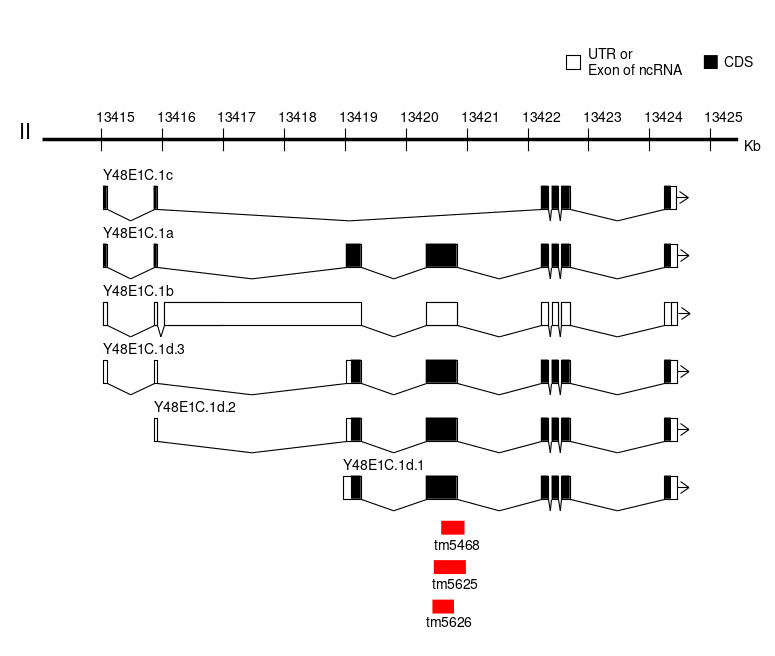
**Figure 1.** Location of the novel alleles

## Description

We report *tm5468*, *tm5625* and *tm5626* as novel deletion alleles of the gene *Y48E1C.1* that is the only ortholog of human calmodulin-lysine N-methyltransferase (CAMKMT)1. CAMKMT encodes an evolutionarily conserved enzyme class I protein methyltransferase that acts in the formation of trimethyllysine in calmodulin for calcium-dependent signaling2. CAMKMT mutation is associated with Hypotonia-cystinuria syndrome in human2,3. The alleles were isolated from the comprehensive screening of gene deletions generated by TMP/UV4. In the screening, all the alleles were detected by nested PCR using the following primer sets, 5’- TCAAGCCACGCCCACACTTA-3’ and 5’- GAAGGCATACAGTGGGGGTA-3’ for the first round PCR and 5’- CGCCCACACTTAATGGTTAT-3’ and 5’- GGGCAGTGTAGGGATACTGT-3’ for the second round PCR. By Sanger sequencing, the 30 bp flanking sequences of the alleles *tm5468*, *tm5625* and *tm5626* were identified as AATCCTTCACACACCACAACAGAAATCCTA – [384 bp deletion] -CGAGGTCACGCCCACACATTGGGCGGAGTT, CCGATGCTCCGTGCTGCTCCAAGTGCTCCG – [627 bp deletion + 9 bp insertion (TAATCTTGT)] – AGTACTCCTACAGTATCCCTACACTGCCCC, and AAAAAAGGATGACGTCACAGTTGCTCCGAT – [256 bp deletion] – ACGCCGATTCGGCAGCCGAATGATCTACAG, respectively. Based on the information about the splicing isoforms of Y48E1C.1 (WormBase, http://www.wormbase.org, WS259), the forth exon of *Y48E1C.1a, Y48E1C.1b* (annotated as non cording RNA) and the second exon of *Y48E1C.1d* transcripts are deleted in *tm5468*, *tm5625* and *tm5626*(Fig. 1). Presumably, all of the alleles do not affect *Y48E1C.1c*. According to information of protein in Wormbase, this exon contains a predicted some motif, suggesting hypothetical functional deficiency of *Y48E1C.1aY48E1C.1b*, and *Y48E1C.1d* in the deletion mutants. In addition, these alleles are expected to be usable for comparing functions among the isoform c and the other isoforms. However, no visually obvious phenotypes (Let, Unc, and Dpy) were observed in *tm5468*, *tm5625* and *tm5626*.

## Reagents

FX05468 *Y48E1C.1* *(**tm5468**)* II (Not outcrossed)

FX05625 *Y48E1C.1* *(**tm5625**)* II (Not outcrossed)

FX05626 *Y48E1C.1* *(**tm5626**)* II (Not outcrossed)
